# Primary hyperparathyroidism in pregnancy: a case report and review of the literature

**DOI:** 10.3389/fmed.2026.1912947

**Published:** 2026-08-26

**Authors:** Li Hao, Yalin Chen, Yumei Lu, Rui Wang

**Affiliations:** Department of Thyroid Surgery, Qingdao Central Hospital, University of Health and Rehabilitation Sciences, Qingdao, Shandong, China

**Keywords:** case report, hypercalcemia, pregnancy, primary hyperparathyroidism, treatment

## Abstract

Primary hyperparathyroidism during pregnancy represents an exceedingly rare clinical entity. Its early manifestations are frequently subtle and easily masked by physiological adaptations of gestation, frequently leading to diagnostic delay. Persistent hypercalcemia carries substantial hazards to both maternal and fetal wellbeing, while relevant clinical literature remains limited. Herein, we present an advanced maternal age patient with recurrent pregnancy loss complicated by second-trimester persistent nausea and vomiting. Serum biochemistry demonstrated marked hypercalcemia and elevated parathyroid hormone, cervical ultrasonography identified a hypoechoic nodule within the right parathyroid gland. The combined biochemical and imaging findings were consistent with pregnancy-associated primary hyperparathyroidism. Following medical correction of hypercalcemia, the patient successfully underwent parathyroidectomy under general anesthesia. However, at 2 months postpartum, she developed acute abdominal pain due to renal calculi and subsequently underwent lithotripsy. In summary, primary hyperparathyroidism in pregnancy is relatively rare, however, vigilant monitoring of serum calcium levels is imperative throughout the gestational period. Management strategies should be individualized based on gestational age and clinical severity to optimize maternal and fetal outcomes. For severe cases requiring surgery, precise preoperative localization of parathyroid lesions is essential. Notably, the second trimester is considered the optimal window for surgical intervention.

## 1 Introduction

Primary hyperparathyroidism (PHPT) is a clinical syndrome characterized by the autonomous overproduction of parathyroid hormone (PTH) from the parathyroid glands. This leads to disorder in calcium and phosphorus metabolism, subsequently affecting multiple organ systems, including the renal and skeletal systems (1). Due to the non-specific nature of its early clinical manifestations, accurate epidemiological data on PHPT remain limited both domestically and internationally. PHPT during pregnancy is particularly uncommon, with reported rates of less than 1% of all PHPT cases in Western countries (2). Date from a single-center study in China indicate an incidence of approximately 0.38% (3). Uncontrolled PHPT not only predisposes pregnant women to hypercalcemic crisis, osteopathy, renal impairment and eclampsia, but also elevates fetal risks including intrauterine growth restriction, miscarriage, and neonatal morbidities such as low birth weight, hypocalcemic seizures and even death (4, 5). Epidemiological data indicate that the incidence of maternal complications in pregnant patients with PHPT may reach 68.7%, with the fetal complication rate as high as 80% (6). In this case, a patient with PHPT was not diagnosed until the second trimester due to atypical symptoms, however, a favorable pregnancy outcome was ultimately achieved through multidisciplinary collaboration.

## 2 Case description

**FIGURE 1:**
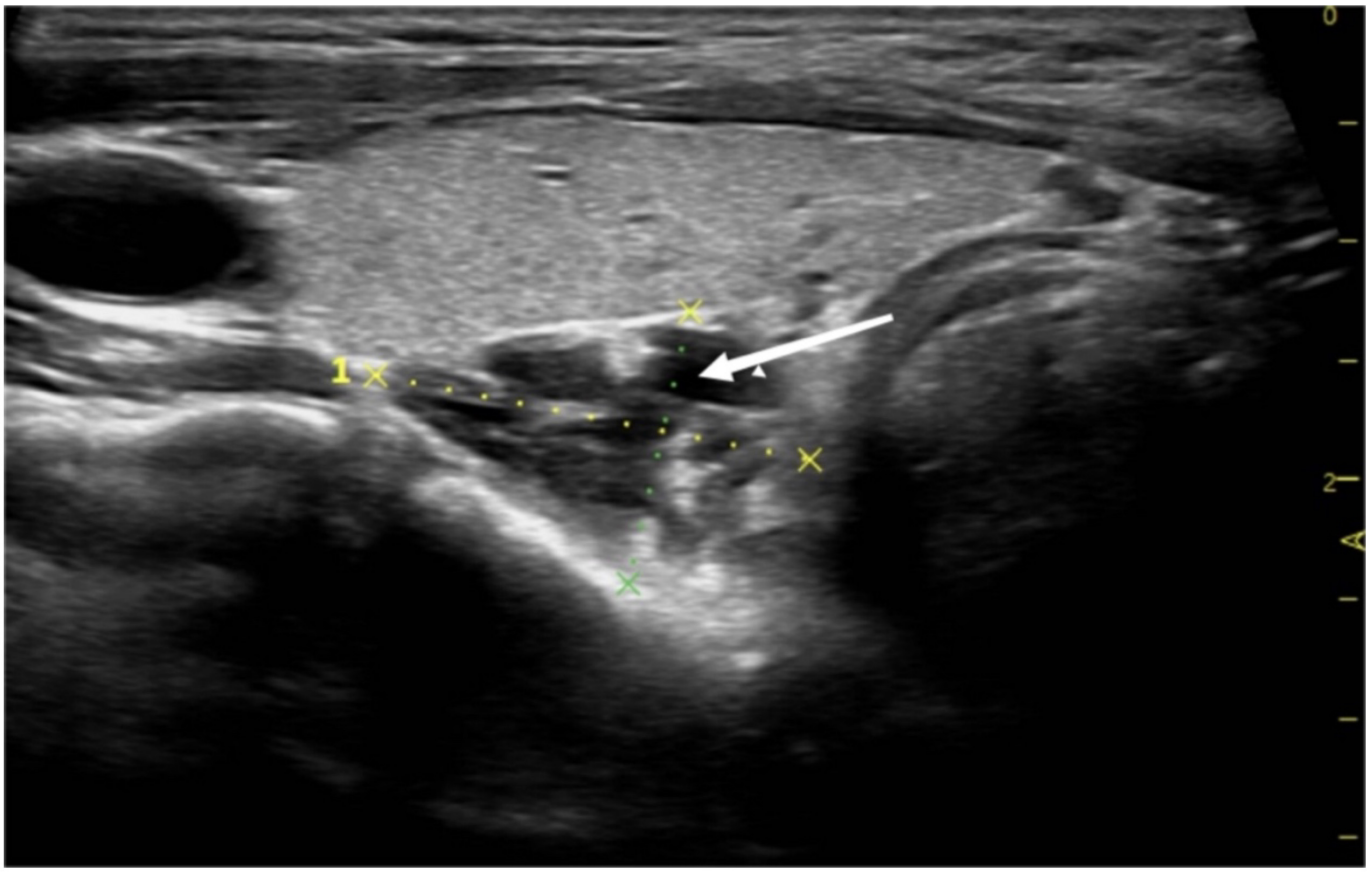
A heterogeneous hypoechoic mass (1.9 × 1.2 cm) with irregular shape, indistinct borders, and abundant internal blood flow was observed in the lower portion of the right thyroid lobe (arrow).

**FIGURE 2:**
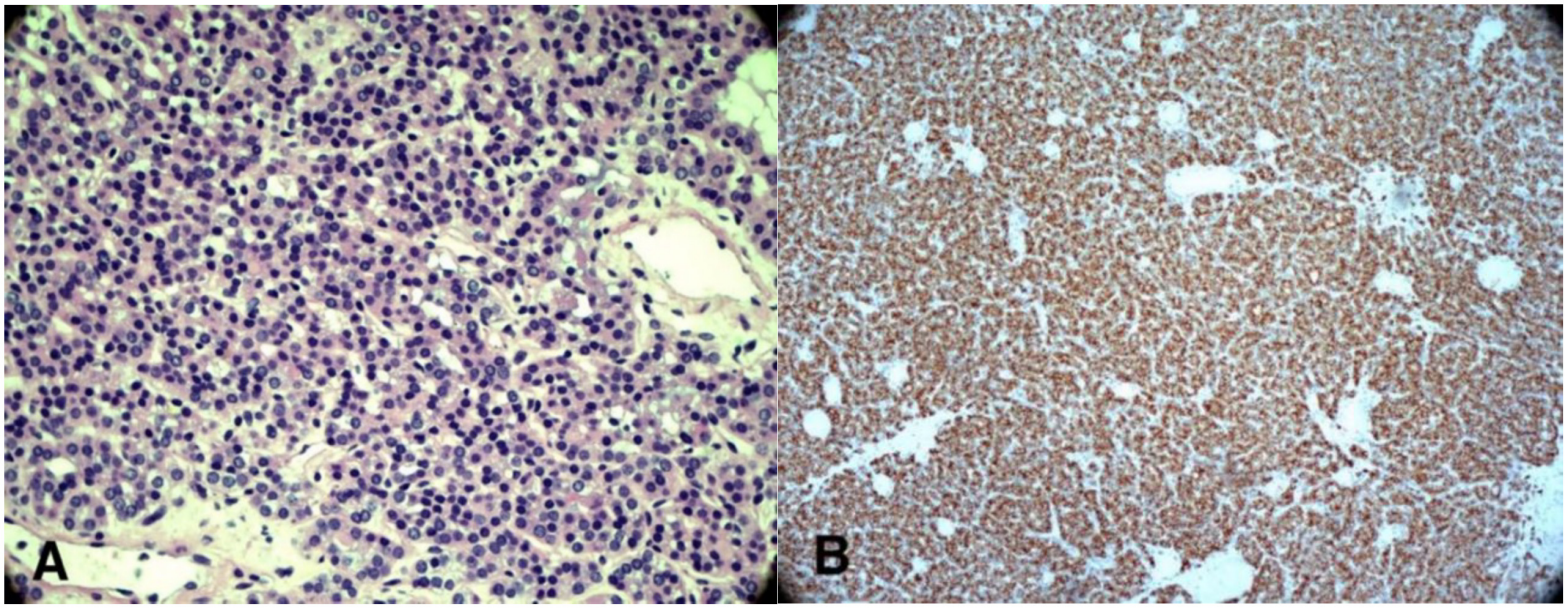
The microscopic images. **(A)** Under 40 × magnification, parathyroid adenomas consist of proliferating chief cells, admixed with oncocytic and clear cells, arranged in sheets, nests, or acini. **(B)** Positive reaction to PTH on immunochemical stains.

A 42-year-old woman presented to the obstetric outpatient clinic with persistent nausea and vomiting of over one month’s duration. Her gestational age was estimated to be 15 weeks, and hyperemesis gravidarum was subsequently diagnosed. Her past medical history was unremarkable, with no history of psychiatric illness. Additionally, there was no family history of hypercalcemia, hypocalciuria, parathyroid surgery, or endocrine tumors. The obstetric history was documented as gravida 5, para 1, abortion 3 (G5P1A3). On admission, vital signs were stable (temperature 36.5 °C, pulse 90 bpm, respiratory rate 20/min, blood pressure 110/79 mmHg). Physical examination revealed a lean body habitus (weight 44 kg, height 155 cm, BMI 18.3 kg/m^2^). Peripheral perfusion was preserved, as evidenced by warm extremities and a capillary refill time < 2 s, however, the skin was dry with poor turgor, consistent with moderate dehydration, and the patient appeared mildly lethargic. Electrocardiography showed sinus rhythm with second-degree atrioventricular block. Notable laboratory findings were as follows: Serum total calcium: 4.49 mmol/L (normal range: 2.11–2.52 mmol/L), the corrected calcium: 4.638 mmol/L. Parathyroid hormone (PTH): 621 pg/mL (normal range: 15–75 pg/mL), Serum magnesium: 0.69 mmol/L (normal range: 0.75–1.02 mmol/L), Serum phosphorus: 0.65 mmol/L (normal range: 0.85–1.51 mmol/L), 25-Hydroxyvitamin D: 18.8 ng/mL (normal range: 30–100 ng/mL), Serum albumin: 32.6 g/L (normal range: 40–55 g/L). Serum creatinine was 64 μmol/L, and the estimated glomerular filtration rate (eGFR) was 106 mL/min/1.73 m^2^. Color doppler ultrasonography of the parathyroid glands revealed a hypoechoic mass in the right parathyroid region, with a size of approximately 1.9 × 1.2 cm (Figure 1). Upon admission, a multidisciplinary team (obstetrics, endocrinology, anesthesiology, and thyroid surgery) urgently convened. A diagnosis of PHPT in pregnancy and hypercalcemia was established based on symptoms, physical findings, and ancillary tests. Due to the significant risks posed by severe hypercalcemia to both mother and fetus, aggressive calcium reduction therapy is required, followed by expeditious surgery. Following volume expansion (2,500–3,000 mL) combined with low-dose furosemide (40 mg daily) to promote urinary calcium excretion, the serum calcium level decreased to 3.49 mmol/L (corrected calcium: 3.638 mmol/L), and the patient underwent resection of the parathyroid lesion under general anesthesia at a gestational age of 15^+2^ weeks. Intraoperative frozen section analysis confirmed a parathyroid adenoma (Figure 2), and the 10-min post-excision PTH level was 43.8 pg/mL, confirming biochemical cure. The resected pathological specimen measured 4 × 1.5 × 1.5 cm. To prevent threatened miscarriage, intravenous magnesium sulfate was administered at a dose of 10 g per day on the day prior to and the day following surgery. On the first day after surgery, the patient’s serum calcium level was 2.98 mmol/L (corrected calcium: 3.128 mmol/L), which remained above the normal reference range, however, the PTH level was only 1.49 pg/mL, accordingly, the patient received intravenous calcium gluconate (4–6 g/day) along with intramuscular vitamin D2 (200,000 IU) during hospitalization. Six days after surgery, the patient’s blood calcium returned to normal and was discharged. After that, therapy was converted to oral calcium carbonate 3 g/day (equivalent to 1,200 mg elemental calcium) and calcitriol (1 μg/day). PTH returned to normal 1 month after the surgery (Table 1). At 39^+6^ weeks of pregnancy, the patient underwent cesarean section, delivering a female infant weighing 3.1 kg. Postnatally, the infant’s serum calcium and PTH were normal, and no hypocalcemia or other adverse clinical manifestations were observed. At 2 months postpartum, she presented with acute right abdominal pain and hematuria. CT revealed a 6-mm renal calculus with mild hydronephrosis. After failed conservative management, she underwent successful extracorporeal shock wave lithotripsy and was discharged. At follow-up, both mother and neonate were healthy.

**TABLE 1:** Temporal evolution of laboratory tests before and after parathyroidectomy.

Time	At admission	On day-1	POD 1	POD 25	POD 39	Reference range
PTH (pg/mL)	621↑	424↑	1.49↓	10.2↓	21	15∼75
Total serum calcium (corrected calcium)* (mmol/L)	4.49 (4.638)↑	3.49 (3.638)↑	2.98 (3.128)↑	2.43	2.3	2.11∼2.52
Phosphate levels(mmol/L)	0.56↓	0.56↓	0.78↓	1.1	0.95	0.85∼1.51
25-hydroxyvitamin D (ng/mL)	18.8↓	–	–	26.1↓	29.1↓	30∼100

PTH, parathyroid hormone; POD, postoperative day; “–,” indicates undetected;

“*,” corrected calcium (mmol/L) = measured calcium (mmol/L) + [40−measured albumin (g/L)] × 0.02, albumin was not monitored after discharge, so calcium values were not corrected.

## 3 Discussion

### 3.1 Clinical symptoms that may be overlooked

Pregnant patients with PHPT may present with intractable gastrointestinal symptoms (including nausea and vomiting), progressive muscle weakness, and weight loss during the first trimester. These clinical features are frequently misdiagnosed as hyperemesis gravidarum or functional gastrointestinal disorders (7). As the disease progresses, affected patients typically present with skeletal and renal complications, such as fibrous cystic osteopathy, osteoporosis, pathological fractures, bone pain, nephrolithiasis, hematuria and even renal failure (4). A small subset of patients with PHPT may also present with rare complications such as acute pancreatitis or life-threatening hemorrhage caused by parathyroid adenoma rupture (8, 9). In the present case, the patient presented with persistent gastrointestinal symptoms after the 13th week of gestation, coupled with laboratory evidence of hypercalcemia, hypomagnesemia and hypophosphatemia, these findings indicated that the metabolic disorder had exceeded the physiological compensatory capacity of the maternal body during pregnancy.

### 3.2 A challenging diagnosis

In non-pregnant individuals, PHPT is typically characterized by elevated serum calcium accompanied by elevated or inappropriately normal PTH levels. However, during pregnancy, physiological adaptations—including placental calcium transfer, hemodilution, and enhanced renal calcium excretion—lead to a physiological decline in total serum calcium concentrations (5). As a result, total calcium measurement alone does not accurately reflect the biologically active ionized calcium fraction and may obscure or delay the diagnosis of PHPT in pregnancy. Studies indicate that the misdiagnosis rate for PHPT during pregnancy can exceed 50% (10). Conversely, ionized calcium and albumin-corrected calcium levels remain relatively stable; hence, their measurement — ideally ionized calcium — is essential for accurate biochemical assessment. Before a diagnosis of PHPT can be confirmed, however, it is important to rule out familial hypocalciuric hypercalcemia (FHH) and secondary hyperparathyroidism (SHPT) (11). FHH is characterized by lifelong, asymptomatic mild hypercalcemia, normal to mildly elevated PTH, and low urinary calcium excretion. The calcium-to-creatinine clearance ratio (CCCR), derived from simultaneous measurements of calcium and creatinine in serum and a 24-h urine specimen, serves as a useful tool for differential diagnosis. A CCCR of less than 0.01 is highly suggestive of FHH, while a value exceeding 0.02 supports a diagnosis of PHPT (12). Conversely, SHPT, often resulting from chronic kidney disease or vitamin D deficiency, typically presents with elevated PTH and normal or low serum calcium levels (13).

It is reported that approximately 85% of sporadic PHPT cases are caused by solitary parathyroid adenomas, around 15% by multiple parathyroid adenomas or parathyroid hyperplasia, and only about 1% by parathyroid carcinoma (14). After the clinical diagnosis of PHPT in pregnancy is established, localization imaging is warranted. Literature reports that high-resolution ultrasonography offers a sensitivity as high as 95.45% for parathyroid lesion localizationlocalization (15). Due to radiation exposure, high cost, and false-positive risks, CT, ^99_m^Tc-MIBI scintigraphy, and MRI should generally be avoided. Their use should be restricted to cases of failed preoperative localization or high suspicion of malignancy (16).

### 3.3 Treatment strategy

#### 3.3.1 Medical management of hypercalcemia

The primary therapeutic objective in managing hypercalcemia is to correct hypovolemia and to enhance calciuresis by increasing the glomerular filtration rate. Fluid management should be individualized: oral rehydration is preferred for pregnant women with mild-to-moderate hypercalcemia, whereas intravenous normal saline is required in severe cases. During fluid resuscitation, rigorous monitoring of respiratory status, pulmonary rales, jugular venous distension, and urine output is essential to prevent volume overload and its complications, including pulmonary edema and heart failure (17). Furosemide can promote urinary calcium excretion and thereby lower serum calcium levels. However, its use during pregnancy carries risks of reduced uteroplacental perfusion, fetal growth restriction, and hypokalemia. Therefore, it is not recommended for routine use and should be reserved for pregnant women with severe hypercalcemia, heart failure, or renal insufficiency (18).

Cinacalcet, a calcimimetic agent, lowers serum calcium by activating calcium-sensing receptors on the parathyroid gland, thereby inhibiting PTH secretion, and by reducing renal calcium reabsorption (19). Denosumab exerts potent anti-resorptive effects through specific inhibition of RANKL, which blocks osteoclast formation and survival (20). Bisphosphonates, in contrast, reduce bone turnover by decreasing both the number and activity of osteoclasts (21). Although all three agents are effective in lowering serum calcium, they cross the placental barrier and have adverse effects on the fetus, therefore their use is not recommended during pregnancy (5). Although literature reports describe the efficacy of cinacalcet in treating PHPT in pregnancy, its use is recommended only in cases of refractory severe hypercalcemia (22). Calcitonin lowers serum calcium by suppressing bone resorption and intestinal calcium absorption while promoting renal calcium excretion (23). It does not traverse the placental barrier, and available evidence suggests that subcutaneous administration at 4–8 IU/kg every 12 h is safe during pregnancy. However, its clinical utility is limited by the rapid development of tachyphylaxis, which leads to a progressive loss of efficacy over time (18).

#### 3.3.2 Surgical intervention

##### 3.3.2.1 Timing of surgery and surgical approach

Serum calcium levels exceeding 3.5 mmol/L may potentially lead to maternal coma, placental vasoconstriction, fetal arrhythmia, or even fatal outcomes (4, 5). Parathyroidectomy is recommended when serum calcium exceeds 2.85 mmol/L or evidence of end-organ damage is present (24). Parathyroidectomy is preferably performed during the second trimester (13–27 weeks), as surgery in the first trimester carries increased risks of teratogenicity and spontaneous abortion, whereas intervention in the third trimester is associated with a higher likelihood of preterm birth. In this case, unilateral parathyroid exploration with adenoma enucleation was performed, avoiding extensive bilateral exploration that would otherwise have prolonged the operative time and elevated the risk of recurrent laryngeal nerve injury (25).

##### 3.2.2.2 Perioperative medication management

Literature suggests that hypomagnesemia may exacerbate the risk of osteoporosis and nephrolithiasis in patients with PHPT (26). In this context, the administration of low-dose magnesium sulfate served a dual purpose: correcting the electrolyte imbalance and acting as a functional calcium channel antagonist to inhibit uterine contractility.

Vitamin D deficiency reduces intestinal calcium absorption, leading to hypocalcemia, which in turn stimulates PTH secretion and synthesis. Moreover, deficiency of vitamin D removes the VDR-mediated transcriptional suppression of the PTH gene, thereby directly enhancing PTH production. Additionally, parathyroid cells can locally convert 25-hydroxyvitamin D to its active form, when vitamin D is deficient, this local autocrine production is diminished, further contributing to PTH elevation. Studies have shown that PHPT patients with coexisting vitamin D deficiency are at significantly higher risk of severe postoperative hypocalcemia and hungry bone syndrome (27); additionally, vitamin D supplementation does not raise calcium levels when serum calcium is below 3.5 mmol/L (28). In individuals with 25-hydroxyvitamin D deficiency or insufficiency (levels < 20 ng/mL), supplementation with native vitamin D like cholecalciferol at a daily dose of 1,500–2,000 IU is recommended (5). In contrast, active vitamin D analogues such as alfacalcidol and calcitriol should not be used for replenishing vitamin D stores, because they do not increase 25(OH)D levels and may pose a greater risk of hypercalcemia.

Following the resection of pathologic parathyroid gland(s), PTH levels typically plummet, reaching a nadir on the 3rd or 4th postoperative day, which consequently precipitates hypocalcemia (29). Therefore, postoperative management should include a high-calcium diet, intravenous and oral calcium supplementation, and vitamin D therapy to enhance calcium absorption. For pregnant patients with PHPT, the recommended postoperative elemental calcium dose is 0.5–1 g/day following single-gland surgery, and 1–3 g/day in cases of multi-gland or repeat surgery, The doses of calcium and vitamin D supplements should be individualized, with close monitoring of serum calcium and PTH levels (30).

#### 3.3.3 Thermal ablation therapy

Thermal ablation has recently emerged as an effective and safe treatment for hyperparathyroidism, with reports documenting successful applications even in pregnant patients (31). However, variations in operator expertise and the inherent anatomical proximity of parathyroid lesions to the recurrent laryngeal nerve may increase the risk of nerve injury or result in incomplete ablation.

#### 3.3.4 Dialysis therapy

While hemodialysis can achieve rapid correction of hypercalcemia, both hemodialysis and peritoneal dialysis pose inherent risks to both the mother and the fetus during pregnancy (32). In addition, the literature on the use of dialysis for hyperparathyroidism in pregnancy is sparse, and robust evidence is lacking. Consequently, dialysis is not considered a routine treatment option for this condition. Instead, it should be reserved as a rescue intervention for severe hypercalcemia that proves refractory to standard medical management.

### 3.4 Follow-up and outcomes

For pregnant patients with untreated PHPT, the abrupt termination of placental calcium transfer postpartum may elevate maternal serum calcium levels and potentially induce a fatal crisis (33). Therefore, close monitoring of postpartum maternal serum calcium levels is essential, and parathyroidectomy should be performed if indicated. Moreover, studies show that maternal serum calcium levels exceeding 2.75 mmol/L significantly increase neonatal hypocalcemia risk (4). Consequently, it is recommended that newborns of mothers with PHPT undergo regular monitoring of serum calcium, PTH, and 25-hydroxyvitamin D levelslevels during the first 1–2 weeks after birth, especially those whose mothers did not receive surgical intervention during pregnancy. In our case, the patient delivered at another hospital, and the neonatal laboratory data were not directly available to us. However, telephone follow-up revealed that the neonate’s serum calcium, PTH, and vitamin D levels were reportedly within normal reference ranges, with no clinical signs of hypocalcemia observed.

The acute episode of renal calculi at two months postpartum indicated persistent organic damage from hypercalciuria. Thus, postpartum surveillance with 24-h urine calcium, renal function tests and ultrasounds is warranted. Furthermore, this case reminds us that pregnant patients with PHPT should undergo renal ultrasound screening for early detection and timely intervention, thereby avoiding acute attacks during pregnancy that could lead to adverse outcomes. In addition, postpartum bone mineral density (BMD) testing is recommended for women who experienced bone and joint pain during the antepartum or postpartum period, in order to monitor for osteoporosis, fracture, and other serious complications associated with hyperparathyroidism (17).

## 4 Conclusion

Primary hyperparathyroidism in pregnancy is a rare condition that threatens both maternal and fetal health, the diagnosis and management depend on early identification of non-specific symptoms, individualized multidisciplinary planning, and appropriate timing of surgery. This case achieved favorable outcomes through precise ultrasound localization, timely surgical intervention during the second trimester, and meticulous perioperative management.

## Data Availability

The original contributions presented in this study are included in this article/supplementary material, further inquiries can be directed to the corresponding author.
